# APOBECs and Herpesviruses

**DOI:** 10.3390/v13030390

**Published:** 2021-02-28

**Authors:** Adam Z. Cheng, Sofia N. Moraes, Nadine M. Shaban, Elisa Fanunza, Craig J. Bierle, Peter J. Southern, Wade A. Bresnahan, Stephen A. Rice, Reuben S. Harris

**Affiliations:** 1 Department of Biochemistry, Molecular Biology and Biophysics, University of Minnesota, Minneapolis, MN 55455, USA; morae010@umn.edu (S.N.M.); nmshaban@umn.edu (N.M.S.); fanun001@umn.edu (E.F.); 2 Masonic Cancer Center, University of Minnesota, Minneapolis, MN 55455, USA; 3 Institute for Molecular Virology, University of Minnesota, Minneapolis, MN 55455, USA; cjbierle@umn.edu (C.J.B.); south003@umn.edu (P.J.S.); bresn013@umn.edu (W.A.B.); ricex019@umn.edu (S.A.R.); 4 Center for Genome Engineering, University of Minnesota, Minneapolis, MN 55455, USA; 5 Department of Pediatrics, Division of Pediatric Infectious Diseases and Immunology, University of Minnesota, Minneapolis, MN 55455, USA; 6 Department of Microbiology and Immunology, University of Minnesota, Minneapolis, MN 55455, USA; 7 Howard Hughes Medical Institute, University of Minnesota, Minneapolis, MN 55455, USA

**Keywords:** APOBEC, DNA cytosine deamination, DNA editing, evolution, innate antiviral immunity, herpesvirus, mutation, restriction factors, ribonucleotide reductase

## Abstract

The apolipoprotein B mRNA editing enzyme, catalytic polypeptide-like (APOBEC) family of DNA cytosine deaminases provides a broad and overlapping defense against viral infections. Successful viral pathogens, by definition, have evolved strategies to escape restriction by the APOBEC enzymes of their hosts. HIV-1 and related retroviruses are thought to be the predominant natural substrates of APOBEC enzymes due to obligate single-stranded (ss)DNA replication intermediates, abundant evidence for cDNA strand C-to-U editing (genomic strand G-to-A hypermutation), and a potent APOBEC degradation mechanism. In contrast, much lower mutation rates are observed in double-stranded DNA herpesviruses and the evidence for APOBEC mutation has been less compelling. However, recent work has revealed that Epstein-Barr virus (EBV), Kaposi’s sarcoma-associated herpesvirus (KSHV), and herpes simplex virus-1 (HSV-1) are potential substrates for cellular APOBEC enzymes. To prevent APOBEC-mediated restriction these viruses have repurposed their ribonucleotide reductase (RNR) large subunits to directly bind, inhibit, and relocalize at least two distinct APOBEC enzymes—APOBEC3B and APOBEC3A. The importance of this interaction is evidenced by genetic inactivation of the EBV RNR (BORF2), which results in lower viral infectivity and higher levels of C/G-to-T/A hypermutation. This RNR-mediated mechanism therefore likely functions to protect lytic phase viral DNA replication intermediates from APOBEC-catalyzed DNA C-to-U deamination. The RNR-APOBEC interaction defines a new pathogen-host conflict that the virus must win in real-time for transmission and pathogenesis. However, partial losses over evolutionary time may also benefit the virus by providing mutational fuel for adaptation.

## 1. Human Herpesviruses

Herpesviruses are abundant in nature, infecting numerous vertebrate species. In humans, herpesviruses are responsible for a variety of clinically important diseases such as cold sores and genital herpes (herpes simplex virus 1 and 2, HSV-1 and -2), chicken pox and shingles (varicella zoster virus, VZV), infectious mononucleosis and several cancers (Epstein-Barr virus, EBV), congenital infections (human cytomegalovirus, HCMV, and HSV-1/2), roseola (human herpesvirus 6A, 6B and 7, HHV-6A, -6B and -7), and Kaposi’s sarcoma and Castleman disease (Kaposi’s sarcoma-associated herpesvirus, KHSV). Infectious virus can be transmitted throughout the lifespan of the host and disease manifestations often recur sporadically causing significant morbidity. Although there are some effective antiviral therapies against herpesviruses, these agents are unable to eradicate the virus and, therefore, infections are lifelong and incurable.

**Figure 1:**
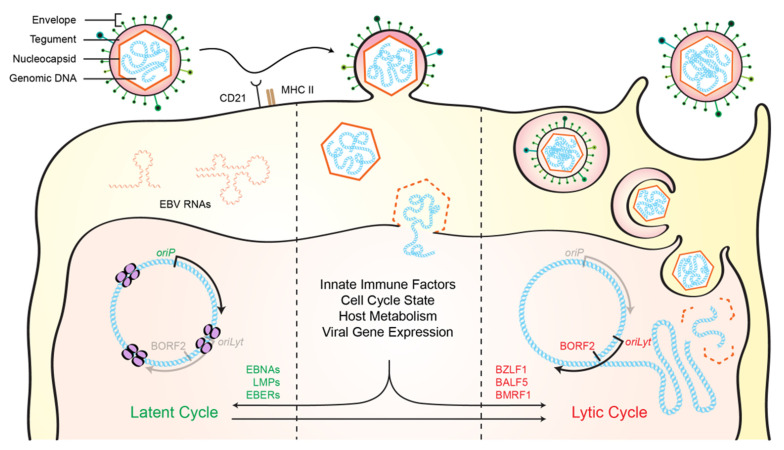
General schematic of herpesvirus replication. A prototypic herpesvirus consists of an outer lipid bilayer with an envelope protein that binds to cellular receptors (e.g., CD21 and MHC-II for EBV) and mediates entry into cells, a tegument layer of viral and host proteins and RNAs used for processes immediately following entry, and an inner icosahedral capsid necessary for nuclear entry and sheltering of the linear double-stranded DNA genome. Several factors, including those listed, determine whether the viral genome enters a latent or lytic replication mode. During latency, herpesviruses express a small subset of proteins and RNAs that combine to maintain the viral genome throughout normal cellular division (e.g., EBV EBNA proteins, LMP proteins, and EBV-encoded small RNAs [EBERs]). Under appropriate conditions, the virus is able to reactivate into the lytic cycle, which results in the production of numerous lytic proteins (e.g., EBV BZLF1, the viral transcriptional activator; BALF5, the viral DNA polymerase; BORF2, the viral ribonucleotide reductase large subunit; BMRF1, the viral processivity factor). New virus production requires viral DNA replication, encapsidation of the viral genome in the nucleus, acquisition of a lipid bilayer envelope in the cytoplasm, and subsequent release from the cellular membrane.

Herpesviruses have linear double-stranded (ds)DNA genomes ranging from 120 to 240 kbp, which code for approximately 80 to 250 distinct proteins [1]. Herpesvirus particles are about 100–200 nm in size with an internal genomic core that is enclosed by an ordered icosahedral capsid [1,2] (Figure 1). Surrounding the capsid is a layer of tegument comprised of viral proteins and RNAs, many of which are required for the initial steps of genome replication following entry of the virus into the host cell [1,2,3]. Lastly, a lipid bilayer envelope surrounds the tegument, containing viral glycoproteins that mediate virus attachment to cell surface receptors and entry into cells [1,2,3].

A major characteristic of herpesviruses is their ability to establish latency inside host cells and switch to a lytic cycle to produce virions for new infections (Figure 1). Several features of the viral latency and lytic replication mechanisms are shared among herpesviruses [1,2]. First, upon infection of a new cell, the linear dsDNA viral genome enters the nucleus, circularizes, and forms a stable extrachromosomal episome, which undergoes methylation, histone modification, and chromatinization [1,2,4]. This process serves to disguise the viral genome as cellular DNA [5] and determines whether the initial infection adopts a latent or lytic replication mode [6,7]. Latency is characterized by limited expression of viral genes to avoid triggering adaptive immune responses. If the host cell divides, viral episomal DNA is replicated in a manner that enables segregation into both daughter cells. EBV, for instance, uses EBNA1 to recruit host DNA replication machinery to the viral replication origin (*oriP*) [1,8]. 

Second, all herpesviruses are able to undergo lytic replication, either during primary infection or by switching from latency to a lytic replication mode (a process termed lytic reactivation [1,2,3]). The molecular mechanisms that govern this switch are not well understood, but a variety of environmental triggers have been implicated including stress, immunosuppression, and UV damage [1,2,4,9]. The lytic replication mode is characterized by coordinated expression of a large number of viral gene products that facilitate genomic DNA replication, particle assembly, and virion release. Viral DNA replication and genome encapsidation occur in the cell nucleus, followed by a complex exit pathway resulting in final envelopment in a cytoplasmic vesicle and egress to the surface of the cell [1,3,10,11]. Many of the conserved herpesvirus proteins are involved in some aspect of viral DNA replication (e.g., ribonucleotide reductase to produce deoxy-nucleosides, ATPase, dUTPase, and thymidine kinase to alter nucleoside pools, uracil DNA glycosylase for repair, and proofreading DNA polymerase for replication) [1].

Herpesvirus mutation rates have been estimated to be nearly as low as those of human chromosomal DNA ([12,13,14]; reviewed by [15]). These low rates have been attributed largely to high-fidelity DNA replication and repair proteins and to the biasing of cellular nucleic acid metabolism to favor viral DNA replication. For instance, herpesviruses are estimated to make only one mistake per 10- to 100-million nucleotides replicated, whereas many retroviruses and RNA viruses make one mistake every 1000 to 10,000 nucleotides replicated. These latter viruses utilize error-prone polymerases (reverse transcriptases and RNA replicases, respectively), and are unable to leverage the high-fidelity machinery of the cell. 

Moreover, retroviruses and RNA viruses are also susceptible to cellular mechanisms capable of directly inflicting mutations in viral replication intermediates. For instance, multiple members of the APOBEC family of DNA editing enzymes are capable of catalyzing the conversion of retroviral cDNA cytosines into uracils (C-to-U), which manifest as genomic strand guanine to adenine (G-to-A) mutations (reviewed by [16,17]). Similarly, the ADAR family of RNA editing enzymes catalyzes the conversion of adenines to inosines, which are replicated as a guanine nucleobases (reviewed by [18,19]). Herpesvirus genomes do not typically exhibit C/G-to-T/A mutations characteristic of APOBEC editing and, until recently, this observation was attributed to the protection afforded by chromatinization and the utilization of high-fidelity DNA replication mechanisms.

## 2. Discovery of a Novel Herpesvirus Ribonucleotide Reductase Interaction

A multi-fluorescent HeLa cell system was used to identify herpesvirus proteins capable of inducing cell cycle arrest [20]. Several viral proteins with this property were affinity-purified from 293T cells and subjected to mass spectrometry to identify potential cellular interacting partners and gain mechanistic insights. The EBV ribonucleotide reductase large subunit, BORF2, was identified in this screen and found to reproducibly bind the ssDNA cytosine deaminase APOBEC3B (A3B) [21]. This result was surprising because prior work had shown that A3B expression in 293T cells is nearly undetectable by real-time PCR, immunoblotting, and DNA deaminase activity assays (e.g., [21,22,23]).

Ribonucleotide reductase enzymes (RNRs) catalyze the conversion of nucleoside diphosphates (NDPs) into deoxy-nucleoside derivatives (dNDPs), which can then be converted to triphosphate forms (dNTPs) and used for DNA synthesis by cellular and viral DNA polymerases [24,25,26]. RNRs are therefore essential for all DNA-based forms of life. Not surprisingly, several viruses including herpesviruses encode their own RNRs, which presumably serve to supplement cellular dNTP concentrations to favor lytic replication. RNRs are divided into class I, II, or III based on a requirement for oxygen and different reaction intermediates [25,26]. Similar to most eukaryotes and aerobic eubacteria, herpesviruses such as EBV express a class I heterotetrameric RNR comprised of two large subunits (BORF2) and two small subunits (BaRF1) [24,25].

In addition to dNDP production, viral RNRs may have other functions in lytic replication. For instance, the HSV-1 RNR large subunit, ICP6 (encoded by *UL39*), has the capacity to inhibit apoptosis and necroptosis [27,28,29,30]. The related HSV-2 RNR large subunit, ICP10 (encoded by *UL39*), has similar activities [29,30,31,32]. These alternative functions of ICP6 and ICP10 are mediated by the N-terminal RIP homotypic interaction motif (RHIM) domain and C-terminal RNR large subunit domain, which interact with receptor-interacting kinases (RIP1 and RIP3) and caspase-8, respectively, to prevent necrosome formation and apoptosis. The RNR large subunit of murine CMV (MCMV) has similar anti-apoptotic properties through a weakly related N-terminal extension [33,34]. Interestingly, the RNR large subunit of HCMV, UL45, is a poor inhibitor of Fas-induced apoptosis, but it has the additional capability of inhibiting NF-κB signaling by targeting RIP1 ([35,36]; reviewed by [24]).

## 3. Mechanism for EBV BORF2 Counteraction of A3B

**Figure 2:**
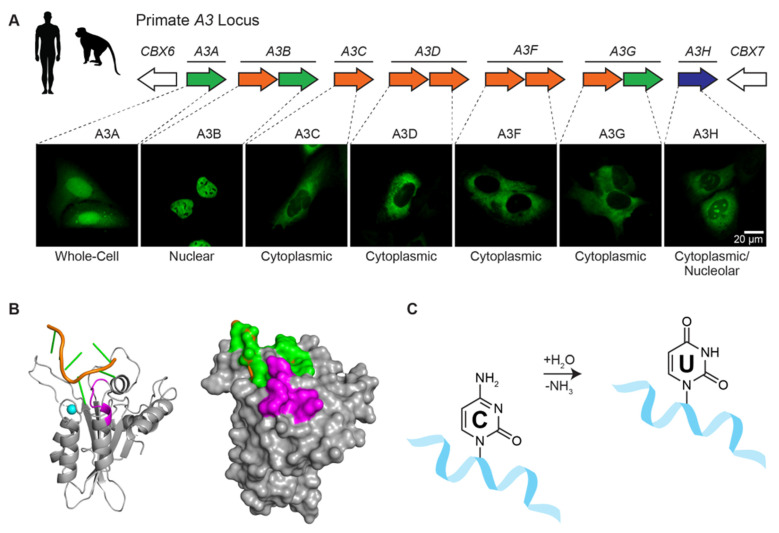
Human APOBEC3 enzymes. (**A**) The human *A3* locus is comprised of seven tandemly arranged genes flanked by *CBX6* and *CBX7* on chromosome 22. Each A3 enzyme has a characteristic subcellular localization (e.g., representative images of U2OS cells expressing the indicated A3-mCherry constructs, colored green to be consistent with Figure 3; A.Z.C. and R.S.H., unpublished). (**B**) Model of the wild-type human A3B catalytic domain bound to ssDNA [43] based on crystal structures of an A3B catalytic domain variant and A3A bound to optimal ssDNA substrates [41]. The deamination preference of A3B for 5’-TC ssDNA substrates (orange and green) is governed by loop 7 residues (purple). A single zinc ion (blue) is located in the catalytic pocket adjacent to the target cytosine nucleobase (green). (**C**) Schematic of the ssDNA cytosine to uracil deamination mechanism, in which A3 enzymes accelerate the hydrolytic replacement of the amine group with oxygen from water.

A3B is one of seven different human APOBEC3 (A3) enzymes—A3A, A3B, A3C, A3D, A3F, A3G, and A3H (reviewed by [16,37,38]; Figure 2A). These single-stranded DNA cytosine deaminases are thought to have overlapping functions in protecting cells from infections by a broad range of viruses including retroviruses such as HIV-1 and HTLV, hepadnaviruses such as HBV, papillomaviruses such as HPV, and polyomaviruses such as JC and BK. Retroviruses are thought to be the principle substrates because they have obligate ssDNA replication intermediates and show clear evidence for APOBEC-catalyzed deamination (e.g., genomic strand G-to-A hypermutation in up to 10% of patient-derived viral sequences [39]). Moreover, lentiviruses such as HIV-1 dedicate a viral accessory protein called Vif to degrade cellular APOBEC3 enzymes and thereby avoid potentially lethal mutagenesis. However, this interaction is not absolute as a significant level of APOBEC mutation may still be able to contribute to immune escape and drug resistance mutations (e.g., [40] and references therein).

Each human A3 is comprised of either one or two globular cytidine deaminase domains. For example, the N-terminal half of A3B is a Z2-type zinc-coordinating deaminase domain and the C-terminal half is a Z1-type domain (shaded orange and green, respectively, in Figure 2A). The N-terminal half of A3B is responsible for localizing the protein to the nucleus and, despite having a conserved zinc-coordination motif, the domain lacks catalytic activity and may serve regulatory roles (nuclear localization shown in Figure 2A). In contrast, the C-terminal domain binds ssDNA using surface-exposed loops adjacent to the zinc-coordinating motif and catalyzes robust ssDNA C-to-U deamination [41,42] (Figure 2B,C).

The modular nature of A3B facilitated mapping of the BORF2 interaction to the catalytic C-terminal domain [21]. Moreover, the loop in A3B responsible for 5’-TC dinucleotide specificity (loop 7) was shown in reciprocal loop swap experiments to be required for the interaction with BORF2 (colored purple in Figure 2B). For instance, BORF2 strongly binds to wild-type A3B in co-immunopreciptation (co-IP) experiments, but not to a chimera with loop 7 residues replaced with those from A3G. Conversely, BORF2 fails to bind to wild-type A3G, but is able to bind a chimeric enzyme with loop 7 residues from A3B. Thus, a single loop region consisting of six amino acids within the A3B catalytic domain is essential for the interaction with BORF2.

As A3B catalytic domain loop 7 residues make multiple contacts with ssDNA substrates, it was hypothesized that BORF2 may function to block A3B from binding to DNA and thereby inhibit its deaminase activity. This idea was tested using purified proteins from *E. coli* and performing in vitro ssDNA C-to-U activity assays [21]. Recombinant EBV BORF2 inhibited A3B enzymatic activity in a dose-dependent manner, and near complete inhibition was achieved with equal concentrations of each protein suggesting a stoichiometric interaction. As a control, activity of the related 5’-TC mutating enzyme A3H was not inhibited, even by nearly 10-fold molar excess of BORF2. These biochemical experiments demonstrated that the BORF2-A3B interaction is direct and that tertiary cellular or viral factors are not required. 

Interestingly, the interaction between human A3B and EBV BORF2 occurs independently of the RNR small subunit BaRF1, and it does not require RNR catalytic residues nor the N- or C-terminal ends of the protein [21]. Rather, a deletion analysis indicated that the structural integrity of the conserved core of the RNR large subunit is essential for mediating the interaction with human A3B [21]. Additional studies are clearly required to reveal the atomic details of this direct protein–protein interaction.

Fluorescent imaging studies revealed an additional, unexpected layer of the underlying molecular mechanism [21]. Either transient expression of BORF2 alone or reactivation of latent EBV to the lytic cycle resulted in the robust relocalization of A3B from the nuclear compartment to cytoplasmic aggregates coincident with endoplasmic reticulum markers. These results were recapitulated in a variety of cell types including model 293T and HeLa cells as well as B cells and epithelial cells, which are natural targets of EBV infection. Time-lapse videos showed dramatic relocalization within 24 h of BORF2 expression, and the most pronounced aggregates were often evident during mitosis following nuclear envelope break-down.

CRISPR-mediated deletion of BORF2 confirmed that this single viral protein is required for A3B relocalization following reactivation of EBV to the lytic cycle [21]. Moreover, in cells infected with BORF2-null EBV, nuclear A3B appeared to concentrate in sub-nuclear regions near viral DNA replication sites marked by the viral processivity factor, BMRF1, and EdU for newly synthesized DNA. Viral stocks resulting from BORF2-null lytic reactivation experiments showed 3- to 4-fold lower titers and an additional 3- to 4-fold lower infectivity per unit of produced virus. Moreover, high levels of viral C/G-to-T/A mutations in A3B-preferred 5’-TC motifs were recovered from the same experiments using 3D-PCR, a technique that enriches for hypermutated DNA sequences, as well as by targeted deep sequencing (MiSeq) in independent regions of the EBV genome. Parallel knockdown experiments demonstrated that both the compromised viral infectivity and all of the observed viral C/G-to-T/A mutations are due to A3B. Serendipitously, BORF2 was shown to be dispensable for viral infectivity in an established EBV model system, the B cell line Akata, which harbors a naturally occurring homozygous *A3B* deletion allele. Taken together, these experiments were the first to show that the EBV RNR large subunit has a non-catalytic function in A3B inhibition and relocalization, and that a failure to perform these activities has major consequences for the virus. 

## 4. Conservation of the Herpesvirus RNR-Cellular APOBEC3 Interaction

**Figure 3:**
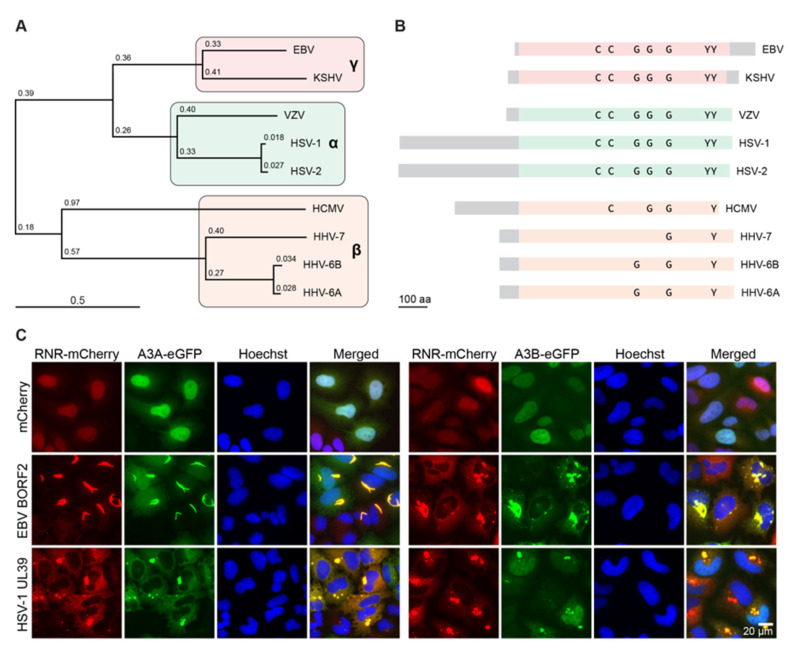
Conservation of herpesvirus RNR activities. (**A**) Phylogram of human herpesviruses based on RNR large subunit amino acid sequences. Gamma-, alpha, and beta-herpesvirus groups are highlighted in red, green, and orange, respectively (EBV BORF2 YP_001129452.1; KSHV ORF61 YP_001129418.1; VZV ORF19 NP_040142.1; HSV-1 ICP6 YP_009137114.1; HSV-2 UL39 YP_009137191.1; HCMV UL45 YP_081503.1; HHV-7 U28 YP_073768.1; HHV-6B U28 NP_050209.1; HHV-6A U28 NP_042921.1). The sequences were aligned using MUSCLE [51] and subsequent phylogeny was generated using neighbor joining tree without distance corrections [52]. (**B**) Schematics of each human herpesvirus RNR large subunit (scale bar = 100 amino acids). Colored regions represent the conserved RNR core domain, and gray regions represent unique N- and C-terminal extensions. The cysteine (C) and tyrosine (Y) residues required for RNR catalytic activity are labeled, with at least one cysteine and one tyrosine lacking from each beta-herpesvirus RNR large subunit. The nucleotide binding domain is also shown with the three essential glycine (G) residues (GxGxxG). (**C**) Representative immunofluorescent microscopy images of HeLa cells expressing BORF2-mCherry or HSV-1 UL39-mCherry (stably) and A3A-eGFP or A3B-eGFP (transiently). The nuclei are stained blue with Hoechst to help distinguish A3A/B relocalization events, which are yellow in the merged images (S.N.M. and R.S.H., unpublished images representative of published work [49]).

The nine herpesviruses that infect humans are classified into three subfamilies: alpha (HSV-1, HSV-2, and VZV), beta (HCMV, HHV-6A/6B, and HHV-7), and gamma (EBV and KSHV) [1] (Figure 3A). Although all herpesvirus subfamilies encode an RNR large subunit, a role for this protein in enzymatic ribonucleotide reduction is not absolute. For instance, none of the beta-herpesviruses have RNR activity due to a deletion that removes the small subunit gene and a mutation that inactivates the large subunit’s catalytic function (reviewed by [24]) (Figure 3B). Alpha-herpesviruses engineered to lack the RNR large subunit showed decreased viral fitness in cell culture experiments as evidenced by lower titers and smaller plaque sizes [44,45,46,47]. Thus, RNR activity is not strictly required for replication of alpha-herpesviruses, at least under model conditions. However, an HSV-1 variant with two point mutations in the RNR large subunit showed severely compromised infectivity in a humanized mouse model, suggesting greater importance in vivo [48].

These observations, along with the recent discovery of the novel A3B counteraction function of EBV BORF2 [21], raised the question of whether or not A3 antagonism is a conserved function of all herpesvirus RNR large subunits. Initial experiments focused on viral RNR large subunit interactions with A3B, which indicated that only gamma-herpesviruses, EBV and KSHV, interacted with A3B [21]. However, after examining a full panel of all seven human A3s in co-IP experiments, these two RNR large subunits were also found to interact with A3A [49]. Moreover, in the same series of experiments, the RNR large subunit of the alpha-herpesvirus HSV-1 was also shown to bind both A3B and A3A [49]. 

To address the relevance of these interactions, A3 relocalization by immunofluorescence microscopy was used as a metric for functional interaction with viral RNR large subunits. Strikingly, there was distinct phenotypic variation in the relocalization of A3B versus A3A (Figure 3C). EBV BORF2 and KSHV ORF61 caused A3B to accumulate in perinuclear globular aggregates that co-localized with endoplasmic reticulum markers, whereas A3A relocalized from a cell-wide/nuclear predominant localization into strikingly linear cytoplasmic structures. It is unclear whether or not the linear A3A aggregates co-localize with the ER or other subcellular structures.

HSV-1 ICP6 overexpression also caused A3B and A3A to relocalize into discrete cytoplasmic structures [49,50]. A clear relocalization phenotype was also evident with wild-type HSV-1 infection, but not with HSV-1 Δ*ICP6* infection. There was also an enhanced relocalization effect using an HSV-1 Δ*ICP4* mutant that only expresses the four immediate early gene products (ICP0, ICP22, UL54, and US12) in addition to ICP6 at higher-than-normal levels, consistent with the idea that only this single viral protein is required for relocalization. However, when measuring the effect of exogenous A3 expression on HSV-1 titers, there was no impact of A3A or A3B over-expression on either wild-type HSV-1 or HSV-1 Δ*ICP6* infectivity [49]. Although this result runs counter to the prediction that A3A or A3B would inhibit HSV-1 replication in the absence of the counter-defense protein, similar to the case with A3B inhibition of EBV Δ*BORF2* replication, alpha-herpesviruses may have additional mechanisms to counter A3-mediated restriction. For example, although A3-mediated uracil lesions may accumulate in the absence of ICP6, repair of these lesions by the viral uracil DNA glycosylase (UL2) could revert these changes and protect genomes from lethal levels of mutation. 

## 5. Evolutionary Perspectives

A powerful way to assess the mutagenic pressure of APOBEC enzymes on virus evolution is to survey for mutational footprints or signatures. This is possible because each APOBEC enzyme has a preferred ssDNA substrate motif. For instance, A3B and A3A preferentially deaminate ssDNA cytosines in 5’-TC motifs, which is evident in biochemical experiments and explained clearly by ssDNA-enzyme co-crystal structures [41,42]. Therefore, over evolutionary time, susceptible viruses are predicted to have an under-representation of A3B/A-preferred 5’-TC substrate motifs and an over-representation of the predominant 5’-TT deamination products. The -2 and +1 nucleobases relative to the target cytosine can also be influential but are not necessary to appreciate this evolutionary perspective.

This general strategy has been applied to herpesviruses specifically [21,53,54], as well as to all publicly available viral sequences [55]. The first analysis of the APOBEC mutation signature in herpesviruses reported a strong under-representation of 5’-TC motifs in VZV, EBV, KSHV and to lesser extents in HSV-1, HCMV, and the other human herpesviruses [54]. This result was confirmed and extended for EBV where 5’-TC motifs were shown to be depleted and 5’-TT motifs enriched in specific genomic regions including the viral replication origin [21]. A more recent paper reported an under-representation of 5’-TC motifs in both EBV and KSHV [53]. The same study also indicated that the EBV origin of replication *oriP* may be a hotspot for A3-catalyzed 5’-TC-to-TT mutation. In addition, AID may contribute to EBV mutagenesis by deaminating preferred 5’-WRC motifs in the viral genomes within B lymphocytes [53,55].

The potential susceptibility of herpesviruses to A3-mediated mutation and restriction has also been assessed in over-expression studies in cell culture [56], where potential viral counterdefense mechanisms may be overwhelmed, and in in vivo murine models [54]. As noted above, human A3B or A3A overexpression has little impact on wild-type or ICP6-null HSV-1 infectivity in cell culture, which may reflect functional redundancy in the A3 counteraction mechanism of alpha-herpesviruses [49]. A3C overexpression, but not A3A, A3G, or AID, appeared to lower HSV-1 titers in cell culture studies [56] but similar results have yet to be reported by other groups. In contrast, human A3B and A3A, but not murine A3 were able to strongly suppress murine gamma-herpesvirus MHV68 spread in cell culture studies [54]. These results suggest that MHV68 may be susceptible to human A3A and A3B, enzymes to which it has never been exposed evolutionarily, and that it has co-evolved to protect itself from the single A3 enzyme of its murine host. As expected, MHV68 replicated equally well in mice with or without the single murine A3 enzyme to which it has likely adapted to counteract [54]. A potential role for the RNR of MHV68 in murine A3 counteraction has yet to be tested.

## 6. Clinical Implications

The discovery of a conserved mechanism of A3 counteraction by herpesvirus RNRs opens up several possibilities for therapeutic intervention. The first lies in disrupting the RNR-A3 interaction as a novel approach for antiviral intervention. Although numerous FDA-approved RNR inhibitors exist for anti-cancer chemotherapy (e.g., hydroxyurea, gemcitabine, gallium nitrate [57,58,59]), their mechanisms are based on either direct enzyme inhibition (e.g., hydroxyurea and gallium nitrate [60,61]) or on indirect alterations of nucleoside pools (e.g., gemcitabine [62]) and, therefore, they are unlikely to perturb the RNR-A3 interaction. Moreover, these chemotherapies may have limited use in the antiviral setting due to human RNR inhibition in non-infected cells and unnecessary toxicity. In comparison, disruption of the viral RNR-A3 interface may generate a more specific drug candidate as there has been no detectable interaction between the human RNR large subunit and A3s [21,49]. This strategy would subsequently allow for uninhibited A3-mediated deamination and hypermutation of the viral genome. Over the course of several viral life cycles within a host, one would predict a compounding effect of A3-mediated hypermutation, leading to eventual extinction of the virus. In vivo experiments in humanized mouse models will be required to test this idea and provide impetus for drug development and human clinical trials.

Whether or not inhibition of the A3-binding domain of the RNR alone is sufficient for clinically beneficial outcomes is still an open question, but some epidemiological data already suggest a role for A3B in limiting the pathogenesis of EBV. Central to any population level study is the fact that an *A3B* deletion polymorphism is common in Southeast Asia and Oceania [63]. Perhaps not coincidentally, these same geographic regions show a higher prevalence of several EBV-associated diseases including chronic active EBV, nasopharyngeal carcinoma, and gastric carcinoma [64,65,66,67]. Even some latency-associated extranodal NK and T-cell lymphomas (NKTL) show geographic differences in incidence, mechanisms of pathogenesis, and disease severity [1,68,69,70]. It is therefore enticing to surmise a potential link between EBV disease incidence and pathogenesis with the allelic distribution of *A3B* deletion polymorphism. If endogenous A3B does indeed play a role in protection from these EBV-associated diseases, it would be logical to assume that A3B would have an even greater effect under conditions where the virally encoded counteraction mechanism is inhibited.

A different approach involves leveraging knowledge of the RNR-mediated A3 inhibition mechanism to develop drugs that specifically block APOBEC enzymatic activity in cancer. Multiple tumor types, including large fractions of breast, head/neck, cervical, bladder, and lung cancers, have accumulated hundreds to thousands of APOBEC-catalyzed mutations (reviewed by [37,71,72,73]). The presence of this mutation process has been associated with poor prognoses including metastasis and drug resistance (e.g., [74,75,76]). The APOBEC family members that are most likely to cause these mutations and contribute to tumorigenesis are A3B and A3A [77,78,79,80,81,82,83]. Therefore, it is reasonable to propose that inhibiting these enzymes would slow down cancer evolution and immune escape, particularly if coupled as an adjuvant therapy to surgery and existing treatments that are often effective but fail due to resistance. This goal could be achieved by designing a herpesvirus RNR-like mimic to inhibit A3B/A deaminase activity in cancer cells and/or to shuttle these enzymes out of the nuclear compartment and away from potential chromosomal DNA substrates. Comprehensive tests of this strategy will require an atomic level understanding of the RNR-A3 interface and a full delineation of the most critical chemical contacts. 

Interestingly, some herpesvirus-based oncolytic therapies have been engineered to lack the viral RNR, presumably for safety and to increase tropism for highly proliferative tumor cells (i.e., cells with abnormally high dNTP concentrations; reviewed by [84,85]). Thus, given knowledge of the RNR-A3 interaction reviewed here, it might be prudent to compare oncolytic viral efficacy in APOBEC-high versus APOBEC-low tumors and ideally also in populations with high frequencies of the naturally occurring *A3B* deletion allele. 

## 7. Concluding Remarks

**Figure 4:**
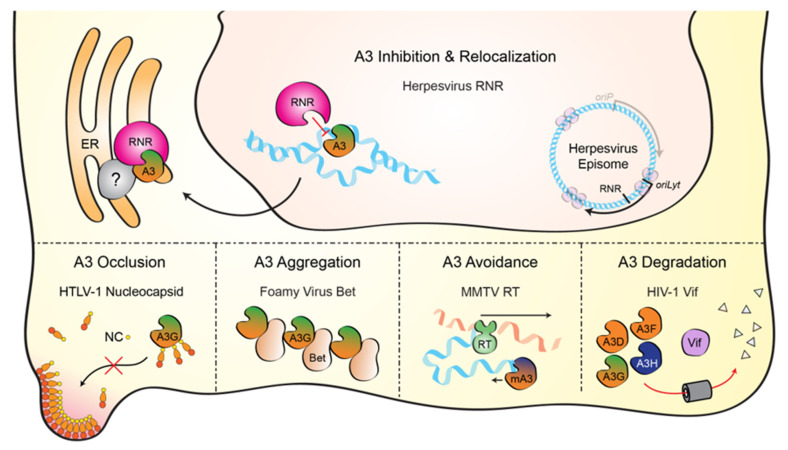
Mechanisms of APOBEC3 counteraction by viruses. **Top**—Schematic of the herpesviral RNR-mediated A3 counteraction mechanism reviewed here. The RNR large subunit (pink) directly binds each A3 enzyme (green/orange), inhibits ssDNA deaminase activity, and protects viral DNA during lytic replication (blue). RNR-A3 complexes accumulate in cytoplasmic aggregates that sometimes associate with the endoplasmic reticulum (ER) and may include as-yet-unidentified cellular factors (gray). **Bottom**—Schematics of additional A3 counteraction mechanisms (left to right): the nucleocapsid component of HTLV-1 Gag blocks A3G from packaging into viral particles, the foamy virus Bet protein promotes A3G aggregation, the murine mammary tumor virus (MMTV) reverse transcriptase synthesizes dsDNA rapidly and thereby limits murine A3 access to ssDNA intermediates, and the Vif proteins of lentiviruses, such as HIV-1, nucleate the formation of an E3 ubiquitin ligase complex that degrades cytoplasmic A3 enzymes (A3D, A3F, A3G, and A3H).

Virus counteraction of cellular A3 enzymes is a critical component of successful viral replication. Several distinct mechanisms have been defined including A3 occlusion (mediated by HLTV-1 nucleocapsid), A3 aggregation (foamy virus Bet), A3 avoidance (MMTV reverse transcriptase), and A3 degradation (lentiviral Vif) (depicted in the bottom portions of Figure 4). Here, we have reviewed the literature, showing that herpesviruses have a unique mechanism to overcome A3-mediated pressures by binding, directly inhibiting, and relocalizing nuclear A3 enzymes to the cytoplasm through the viral ribonucleotide reductase (depicted in the top portion of Figure 4). This process is conserved for at least the human gamma-herpesviruses, EBV and KSHV, as well as the model alpha-herpesvirus, HSV-1. Whether or not beta-herpesviruses also possess an A3 counteraction mechanism is currently unknown, but precedence dictates that any entity with susceptible ssDNA (e.g., DNA replication or transcription intermediates) should be theoretically susceptible to A3 enzymes, including retroviruses, small and large DNA viruses, and sometimes, unfortunately, also cellular chromosomal DNA. Indeed, in a recent pan-viral survey of APOBEC mutation signatures, several types of mammalian DNA viruses including ssDNA and dsDNA viruses showed varying degrees of APOBEC mutation signature [55]. 

One of the continued difficulties in studying the A3 family of paralogs, given high homology between family members, is defining the specific physiologic enzyme (or enzymes) capable of providing innate antiviral immunity to a distinct viral pathogen. For EBV, it is likely that A3B is a physiologic restriction factor as it satisfies several criteria. First, endogenous A3B is expressed in EBV-tropic cell types such as epithelial cells (primary infection) and B cells (latent infection). Second, A3B is the only solely nuclear A3 enzyme and has been shown to co-localize with replicating viral DNA. Third, both A3B and BORF2 are under positive selection, suggestive of ongoing host/pathogen co-evolution [49,86,87]. On the other hand, BORF2 has also been shown to interact with and relocalize A3A, which may be a reflection of sequence homology to A3B and less likely a true physiologic function. Furthermore, the expression profile of A3A suggests that this enzyme may be limited naturally to monocyte and macrophage lineage cell types and is not expressed in B, T, NK, or epithelial cell types (by mining the publicly available RNA expression database FANTOM5 [88]). As such, EBV most likely replicates without being exposed to A3A. Less is known about the RNR-A3 interactions of the other herpesviruses, but it is possible that viruses with broader tropisms, such as KSHV and HCMV, may have had to evolve to be able to effectively neutralize A3A, A3B, and potentially additional A3 enzymes. Regardless, the most important conclusion is that A3 enzymes pose a serious threat to DNA-based viruses. Thus, the herpesvirus RNR-A3 neutralization mechanism can be regarded as the latest exciting chapter in an ever-expanding collection of pathogen–host interaction stories.
